# Conventionally Reared Wallon Meat Lambs Carry Transiently Multi-Drug-Resistant *Escherichia coli* with Reduced Sensitivity to Colistin Before Slaughter

**DOI:** 10.3390/ani14203038

**Published:** 2024-10-21

**Authors:** Delphine Dragon, Wiebke Jansen, Helene Dumont, Laetitia Wiggers, Damien Coupeau, Marc Saulmont, Bernard Taminiau, Benoit Muylkens, Georges Daube

**Affiliations:** 1 Integrated Veterinary Research Unit, Faculty of Science, Université de Namur, Rue de Bruxelles 61, 5000 Namur, Belgiumwiebke@fve.org (W.J.); helene.dumont@unamur.be (H.D.); damien.coupeau@unamur.be (D.C.); 2 Department of Food Sciences, Microbiology, Fundamental and Applied Research for Animal & Health (FARAH), Faculty of Veterinary Medicine, Université de Liège, Avenue de Cureghem 10, 4000 Liège, Belgium; bernard.taminiau@uliege.be (B.T.); georges.daube@uliege.be (G.D.); 3 Federation of Veterinarians of Europe (FVE), Rue Victor Oudart 7, 1030 Brussels, Belgium; 4 Regional Animal Health and Identification Association (ARSIA), 5590 Ciney, Belgium; marc.saulmont@arsia.be

**Keywords:** sheep, meat lamb, colistin, *E. coli*, MDR, ESBL

## Abstract

Major efforts have been made to reduce the use of colistin in livestock since the discovery of the plasmid-borne mobile colistin resistance (*mcr*) gene in *E. coli* a decade ago, to curb the burden of its potential transmission to other bacterial species, spread between animals, humans and the environment. This study explored the longitudinal prevalence and characteristics of colistin-resistant and extended-spectrum beta-lactamase-producing (ESBL) *E. coli* via in vivo fecal and ex vivo carcass swabs from two batches of conventional indoor and organic outdoor Wallon meat sheep from birth to slaughter in 2020 and 2021. Antimicrobial susceptibility testing via broth microdilution revealed that n = 16/109 (15%) *E. coli* isolates from conventional meat lamb fecal samples had a reduced colistin sensitivity (MIC = 0.5 μg/mL) and thereof, n = 9/109 (8%) were multi-drug-resistant *E. coli*, while no resistant isolates were recovered from their carcasses. Sequencing revealed causative *pmrB* genes, indicating that the reduced sensitivity to colistin was not plasmid-borne. While the sample size was small (n = 32), no colistin-resistant and ESBL-producing *E. coli* were isolated from the organic meat sheep and their carcasses, potentially due to the different husbandry conditions. Prudent and judicious antimicrobial use and strict slaughter hygiene remain imperative for effective risk management to protect consumers in a sustainable One Health approach.

## Simple Summary

Public health concerns continue about the spread of colistin resistance, especially after the discovery of the mobile colistin resistance (*mcr*) gene in *Eschierichia coli* a decade ago, which could potentially transfer resistance to other bacteria, posing risks to humans, animals and the environment. This study investigated the presence of colistin-resistant and ESBL-producing *E. coli* in both conventional indoor and organic outdoor Wallon meat lambs from birth to slaughter. Assessing n = 109 fecal samples in 2020 and 2021, 15% of *E. coli* isolates from conventional sheep showed reduced sensitivity to colistin, and 8% of these were multi-resistant. Genetic analysis showed the resistance was due to chromosomal mutations rather than the *mcr-1* gene. In contrast, no resistant *E. coli* was isolated from the n = 32 samples from organic sheep, potentially due to different husbandry practices. The results highlight the importance of careful antimicrobial use and strict hygiene during slaughter to minimize risks to consumers, aligning with the One Health approach.

## 1. Introduction

In 2019, antibiotic-resistant *Enterobacteriaceae*, including pathogenic facultative anaerobic Gram-negative *Escherichia coli*, were estimated to be responsible for the largest number of human deaths attributable to antimicrobial resistance (AMR) due to lower respiratory and thorax infections, bloodstream infections and intra-abdominal infections [1]. Among the World Health Organization (WHO) priority pathogens, carbapenem-resistant and extended-spectrum beta-lactamase-producing (ESBL) *Enterobacteriaceae* were ranked among the critically important bacterial species for which few interventions exist [2]. Colistin, an antibiotic from the polymyxin family, once set aside by human medicine due to its parenteral neurotoxicity and nephrotoxicity, resurged as a last-resort treatment against these multi-drug-resistant pathogens [3]. This resulted in a 67% increase in the consumption of polymyxins (mainly colistin) in human medicine between 2011 and 2020 [4]. In parallel, in the European Union (EU), veterinary colistin sales dropped by 76.5% between 2011 and 2020 though it was widely used in veterinary medicine ever since its discovery for the prevention and treatment of intestinal infections by *Enterobacteriaceae* in livestock [5]. Plasmid-borne ESBL determinants such as *bla*_CTX-M_ genes and the plasmid-borne colistin resistance gene *mcr* (mobile colistin resistance), discovered for the first time in China in 2015, largely favor their ability to be readily transmitted in an inter- or intra-species manner by horizontal transfer [6]. To date, 10 *mcr* variants (*mcr-1* to *mcr-10*) have been identified with multiple subvariants [7]. The spread of the *mcr-1* gene in different *Enterobacteriaceae* has been described worldwide in more than 30 countries on five continents, from animals, and food products thereof, from healthy and infected humans and the environment [3]. Food-producing animals were identified as significant reservoirs of AMR bacteria, exerting a pivotal role at the interface of human–animal–environment interactions. Therefore, harmonized monitoring and reporting of colistin resistance in certain zoonotic and commensal bacteria became mandatory in January 2014 for all EU Member States [8]. The data collected on antibiotic resistance at EU level are harmonized regarding the sampling design, laboratory methodology, reporting and interpretation of resistance of *Salmonella*, *Campylobacter*, *E. coli* and *Enterococcus* in certain populations of food-producing animals and certain products thereof. The third joint inter-agency antimicrobial consumption and resistance analysis (JIACRA) report, issued by the European Medicines Agency (EMA), the European Food Safety Authority (EFSA) and the European Centre for Disease Prevention and Control (ECDC), showed that colistin resistance in indicator *E. coli* isolates from livestock was generally uncommon, with 1.22% of phenotypically resistant isolates for the years 2019 and 2020 [9]. EFSA trend analysis for the period 2014 to 2020 showed a decrease in resistance for broiler and turkey isolates, a stable level for bovine isolates and an increasing trend for porcine isolates [10]. However, neither sheep nor products thereof, such as mutton or raw sheep milk, are included in the sampling plan. In Belgian food-producing animals, colistin resistance in commensal *E. coli* decreased significantly between 2011 and 2018 [11] and in 2021, almost no resistance was observed (1.2% in veal calves, 0.8% in young beef cattle and no resistance in fattening pigs and broilers) [12]. Similarly, data for sheep were not reported in Belgium’s latest One Health report on antibiotic use and resistance in the country [13]. This study explores the prevalence and characteristics of colistin-resistant *E. coli* from two conventional batches and one organic batch of healthy Wallon meat lambs from birth to slaughter and their carcasses over two years.

## 2. Materials and Methods

### 2.1. Sampling In Vivo and Ex Vivo

**Figure 1:**
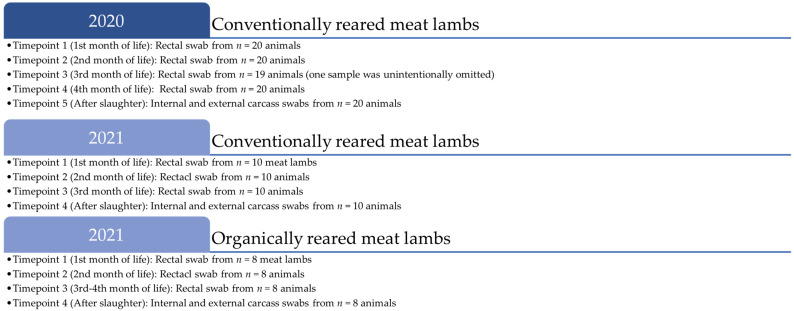
Timeline of in vivo and ex vivo sampling of meat lamb batches with an interval of at least three weeks. An additional in vivo sampling was necessary in 2020 due to slaughter scheduling for market demands.

In 2020 and 2021, each n = 20 and n = 10 lambs, resp., born and bred at the conventional meat lamb farm of the Ovine Research Centre of the University of Namur were selected to be sampled individually. Inclusion criteria were (i) male lambs born within two consecutive weeks, (ii) exclusively fed by their ewe, while exclusion criteria were (i) any atypic physical development or illness during the study period, (ii) the use of antibiotics in their lifetime. Fecal samples were obtained using disposable nylon-flocked sterile FecalSwab™ with 2 mL Cary-Blair Medium (Copan Diagnostics Inc., Murrieta, CA, USA) according to the manufacturer’s instructions four times in 2020 and three times in 2021 during fattening with intervals of at least three weeks (Figure 1). In brief, flocked swabs were inserted 2–3 cm through the rectal sphincter and gently rotated, ensuring that fecal material is visible on the tip of the swab and transferred into labeled FecalSwab™ tubes. Ethical approval was granted under protocol code 20-347 by the Ethical Committee of the University of Namur. In 2020, each 2–3 lambs were scheduled for slaughter over six weeks due to market demands, which made an additional in vivo sampling necessary at three months of age. In 2021, all lambs were scheduled simultaneously for slaughter, which reduced the sampling frequency towards the end. In comparison, eight extensively reared lambs under the EU organic scheme and their carcasses were sampled in 2021 under the same sampling protocol for the presence of ESBL and colistin-resistant *E. coli*.

**Figure 2:**
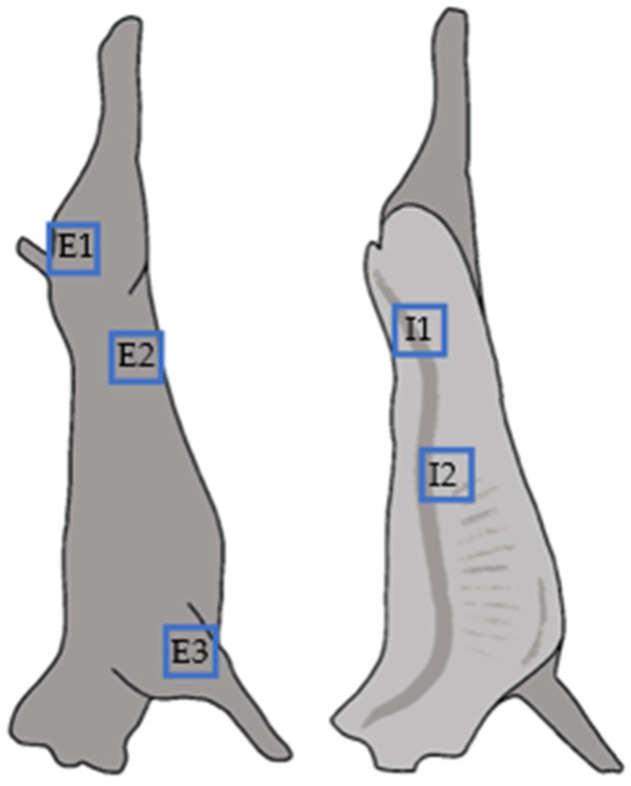
Sampled areas on meat lamb carcasses. The carcass of each lamb was sampled using two swabs: one for the three outer zones (E1–E3), the other for the two internal zones of the two half-carcasses (I1–I2). The surface and areas were determined in accordance with Regulation (EC) n° 2073/2005 [14].

At the time of slaughter, each of the corresponding carcasses were sampled separately on their inner and outer surface. The surface and areas were determined under Regulation (EC) n° 2073/2005 [14], cumulating to each at five different locations for a total area of approximately 50 cm^2^ (5 × 10 cm^2^) per half carcass (Figure 2). Access to the slaughter facilities for supervised sampling was granted by the Belgian Federal Agency for the Safety of the Food Chain. All swabs were transported and stored refrigerated according to the manufacturer’s instructions for max. 72 h until further characterization.

### 2.2. Bacterial Isolation and Identification

A volume of 100 µL from each FecalSwab^TM^ Cary-Blair Medium was plated directly on CHROMID^®^ ESBL chromogenic agar plate (bioMérieux, Craponne, France) in accordance with the manufacturer’s instructions. In parallel, an aliquot of 200 μL out of the 2 mL Cary-Blair Medium from each rectal swab was inoculated in 9 mL broth Brain Heart Infusion (BHI) enrichment broth (Carl Roth GmbH + Co. KG, Karlsruhe, Germany) containing a 10 μg colistin disk (Bio-Rad, Marnes-la-Coquette, France) for 4 to 5 h at 37 ± 0.5 °C under agitation. Of these, 50 μL was streaked on a CHROMID^®^ Colistin-Resistant (COL-R) chromogenic agar plate (bioMérieux, Craponne, France) that was incubated at 37 ± 0.5 °C for 18 to 24 h prior to reading and according to the manufacturer’s instructions. Each one presumptive phenotypic colistin-resistant or ESBL *E. coli* isolate per morphology from each agar plate was streaked on Luria Broth (LB) agar (Carl Roth GmbH + Co. KG, Karlsruhe. Germany) and incubated at 37 ± 0.5 °C for 18 to 24 h. Isolates were stored in a Microbank™ (Pro-Lab Diagnostics, Bromborough, United Kingdom) at −80 °C until further identification by MALDI-TOF (Matrix-Assisted Laser Desorption Ionization/Time Of Flight) mass spectrometry using the Bruker Biotyper (Bruker Daltonics, Bremen, Germany).

### 2.3. Antimicrobial Susceptibility Testing

Determination of the minimal inhibitory concentration (MIC) for phenotypically resistant *E. coli* isolates was performed by antimicrobial susceptibility testing (AST) via broth microdilution assay in 96-well FRCOL and BOP06F microtiter plates covering nineteen antibiotics (ceftiofur, colistin, gentamicin, florfenicol, tiamulin, chlortetracycline, oxytetracycline, penicillin, ampicillin, danofloxacin, neomycin, trimethoprim/sulfamethoxazole, tylosin, tulathromycin, tilmicosin, clindamycin, sulfadimethoxine, enrofloxacin and spectinomycin) according to the manufacturer’s instructions (Sensititre, Fischer Scientific, Hampton, VA, USA). MICs were interpreted in accordance with the guidelines of the European Committee on Antimicrobial Susceptibility Testing (EUCAST) and “Comité de l’antibiogramme de la Société Française de Microbiologie” [15,16,17]. Isolates were classified as microbiologically susceptible (phenotypically not resistant and MIC below the breakpoint) or resistant (phenotypically resistant and MIC above the breakpoint) for each antibiotic based on their MICs; phenotypically resistant isolates with an MIC below the breakpoint were classified as reduced susceptible. Strains resistant to three or more classes of antimicrobial agents were defined as multi-drug-resistant bacteria. In addition to the broth microdilution, disk diffusion susceptibility was performed for the confirmation of five phenotypic ESBL isolates that showed inconclusive cephalosporin resistance covering a panel of 16 antibiotics (amoxicillin, amoxicillin/clavulanic acid, cefotaxime, cefotaxime/clavulanic acid, cefoxitin, cefquinome, ceftiofur, meropenem, colistin, enrofloxacin, marbofloxacin, kanamycin, florfenicol, gentamicin, tetracycline, trimethoprim/sulfamethoxazole).

### 2.4. DNA Extraction and Sequencing

In a targeted approach, only the identified phenotypical ESBL and colistin-resistant *E. coli* were further characterized. DNA extraction was performed using the DNeasy^®^ blood and tissue kit (Qiagen, Hilden, Germany) and the amount of DNA and purity of samples were determined with the NanoDrop™2000c (Thermo Fisher Scientific, Waltham, MA, USA ) as described earlier [18]. The sequencing libraries were prepared using the Nextera XT library preparation kit (Illumina, Evry, France) and sequenced on a Novaseq Illumina machine (Illumina, Evry, France) using V1.5—300 cycles XP workflow at the GIGA Institute (Université de Liège). Raw read sequences are available in GenBank/SRA under the BioProject PRJNA1149975. Read quality cleaning was performed using fastp v0.23.0 [19] and assembly was performed with Unicycler v0.5.1 with SPAdes v3.14 [20]. The identification of acquired antibiotic resistance genes was performed with the Enterobase v1.2 AMR search design tool [21] and AMRFinderPlus v3.12.8 [22] using the specific *E. coli* scheme.

## 3. Results

In total, 133 rectal swabs and 76 carcass swabs were collected and further characterized (Table S1). The third sample from lamb A8 in August 2020 was unintentionally omitted. None of the in vivo or ex vivo samples from the meat lambs reared under the organic scheme harbored phenotypically presumptive colistin-resistant *E. coli* and no presumptively colistin-resistant *E. coli* was isolated from their carcasses. The 109 rectal samples from conventionally reared Wallon meat lambs harbored a total of 16 *E. coli* (15%) with reduced sensitivity to colistin, thereof n = 9 (8%) ESBL-producing *E. coli*.

### 3.1. Presumptive Colistin-Resistant E. coli

Of the 79 rectal swabs collected from the conventionally reared lambs in 2020, n = 2 samples (2.5%) harbored phenotypically presumptive colistin-resistant *E. coli*, which were confirmed by MALDI-TOF. Both strains were isolated from different animals, one in the second in vivo sampling and the other in the third in vivo sampling, but never before or after in the last in vivo sampling. Both *E. coli* showed a reduced sensitivity to colistin (MIC = 0.5 μg/mL) and a reduced sensitivity to tetracycline (MIC = 4 μg/mL) in broth microdilution AST. No presumptively colistin-resistant *E. coli* was isolated from the carcass samples.

Of the 30 rectal swabs collected from the conventionally reared lambs in 2021, n = 5 samples (16.7%) harbored phenotypically presumptive colistin-resistant *E. coli* and were confirmed by MALDI-TOF as *E. coli*. All strains were isolated from different animals, one in the second in vivo sampling and four in the last in vivo sampling before slaughter sampling, but never before or after. All *E. coli* isolates showed a reduced sensitivity to colistin (MIC = 0.5 μg/mL). No presumptively colistin-resistant *E. coli* was isolated from the carcass samples.

All seven *E. coli* isolates with reduced colistin sensitivity were not resistant to any other antibiotic according to the AST panel used. Sequencing identified two pathovars, four sequence type complexes and two resistance genes conferring resistance against colistin and fosfomycin (Table 1).

### 3.2. Presumptive ESBL-Producing E. coli

Of the 79 rectal swabs collected in 2020, n = 9 samples (11%) showed phenotypic ESBL *E. coli*. Each two distinctive strains were isolated from lamb A4 (first and third sampling) and A20 (first and third sampling). The five remaining strains were isolated from different animals during the third sampling, but never before or after. All isolates presented the typical phenotypic synergy of ESBL production and were resistant to three or more antibiotic classes and therefore classified as multi-drug-resistant (MDR) according to the definition by Magiorakos et al. [23]. In addition, all ESBL isolates also showed a reduced phenotypic sensitivity to colistin with an MIC of 0.5 μg/mL. Two presumptive phenotypic ESBL *E. coli* isolates (n° 7579-39 and 7548-19), which showed a ceftiofur MIC <4 μg/mL and therefore categorized initially as susceptible, were subjected to additional disk diffusion AST. All strains showed an ESBL phenotype with synergy between amoxicillin and ceftiofur and a diameter difference between cefquinone and cefquinone + clavulanic acid greater than 5 mm and were therefore eventually categorized as ESBL [17]. All nine isolates presented phenotypic resistance to non-beta-lactam antibiotics, most commonly against sulfamethoxazole/trimethoprim (n = 9/9, 100%), gentamycin (n = 2/9, 22%) and tetracycline, (n = 2/9, 22%) (Table 2). No phenotypic resistance was observed for meropenem and enrofloxacin. No presumptive ESBL *E. coli* were isolated from the 30 rectal swabs collected from the conventionally reared lambs in 2021, neither from the organically reared lambs nor from any carcass sample.

The identified genes were coherent with the observed ESBL phenotypes apart from isolates no 7579-39 and 7548-19. While these isolates carried *blaTEM-1* and *blaCARB-2*, sequencing revealed that the ESBL phenotype was due to the presence of *blaTEM-52* genes in the remaining isolates. A diversity of additional resistance genes was identified, most commonly the ANT(3″)-Ia family aminoglycoside nucleotidyltransferase gene *aadA2* (n = 9/9, 100%) and the gene *glpT_E448K* (n = 8/9, 89%) encoding for fosfomycin resistance. The lincosamide nucleotidyltransferase gene *Lnu(F)* was detected in seven isolates (78%). The phenotypic tetracycline resistance of isolate 7579-39 corresponds with the detection of the *tet(A)* gene. The gentamycin resistance of isolate 7548-19 corresponds with the detection of the *aac(3)-IId gene*. Seven isolates (78%) carried the *pmrB* gene corresponding to their phenotypically reduced sensitivity to colistin, while two isolates showed a discrepancy between their colistin-resistant phenotype and their genotype. Isolate A10 shows a discrepancy between the ceftiofur-resistant phenotype and its genotype. The phylogenetic analysis showed the absence of phylogroup B2 and the predominance of lineage AxB1 and sequence type (ST) 3594 (n = 7/9, 78%) (Table 3 and Table S2).

## 4. Discussion

This is the first report of the transient fecal carriage of multi-resistant *E. coli* with reduced sensitivity to colistin in Wallon meat lambs, a less scrutinized production sector compared to other livestock species. While European legislation does not require AMR surveillance in sheep or products thereof, such as mutton or raw milk cheese, these food items are often consumed undercooked or raw, leading to a potential public health risk.

### 4.1. Transient Fecal Carriage of Multi-Resistant E. coli with Reduced Sensitivity to Colistin

There is a growing body of evidence that sheep in Europe and beyond are a reservoir for ESBL and colistin-resistant *E. coli* [24,25,26,27,28,29]. In our study, Wallon meat lambs carried transiently ESBL *E. coli* with a reduced sensitivity to colistin. Transient shedding of *E. coli* was shown before, exemplified by a study on intermittent excretion of stx-negative *E. coli* O26 from orally inoculated 6-week-old lambs beyond day 30 and just above the detection limit [30]. In experimentally inoculated calves, intermittent fecal shedding of *E. coli* O157:H7 did not lead to isolation from sites outside the alimentary tract [31]. In China, Hu et al. reported low levels of colistin-resistant *E. coli* carrying the mcr-1 gene in sheep, with n = 9 positive isolates obtained from 246 fecal samples [32]. However, a total of 29 fecal samples from French sheep harbored 11 *E. coli* with an MIC > 2 μg/mL, including 6 isolates carrying the mcr-1 gene [33]. Dantas Palmeira demonstrated a high proportion of ESBL *E. coli* in Portuguese sheep feces, with one *mcr*-positive isolate [25]. Compared to our results, the French and Portuguese results show colistin-resistant *E. coli*, which could be related to their colistin use. However, Belgian national measures to limit colistin use in livestock led to a decrease in colistin-resistant *E. coli* in livestock and humans, which is in line with our results [13]. However, all *E. coli* isolated from our study showed a reduced sensitivity to colistin, while this antibiotic was neither used previously at the sampled farm nor in the sampled batches. It was shown before by the third JIACRA report that colistin resistance in indicator *E. coli* and polymyxin consumption in livestock for the time periods 2014 to 2018 in the majority of European countries had statistically significant positive associations [9]. Yet, the majority of European veterinarians specializing in sheep and goats in Europe ceased using colistin and veterinary colistin sales dropped by 76.5% between 2011 to 2020 [5]. Therefore, the limits imposed in the EU on the use of colistin livestock may not be sufficient to effectively mitigate colistin resistance due to cross-resistance with other antibiotics as reduced colistin sensitivity in ovine isolates regularly occurs simultaneously with resistances to other antibiotics [34]. The genetic characterization of the isolates from our study revealed that the reduced colistin sensitivity was mainly conferred by the pmrB gene, which is part of the PmrAB chromosomal two-component system. Point mutations occurring in pmrA and pmrB genes lead to pmrAB expression increase and changes in the outer membrane to reduce the membrane uptake of colistin responding to environmental stimuli as a response regulator to ion presence and pH changes [35]. It was shown that nonsynonymous polymorphisms in the PmrAB two-component system of S. enterica and *E. coli* isolated from chicken eggs and swine feces led to colistin-resistant strains due to the selective pressure of colistin-inducing genetic mutations in PmrA, PmrB, PhoP, PhoQ, MgrB and PmrD [36]. While colistin was never used on this farm or in these lambs, collaborative efforts should remain a priority to further reduce, in particular, colistin use and in general, the need to use antibiotics in livestock.

ESBL-producing *E. coli* have been reported in sheep in and outside Europe with proportions ranging from 1% to 90% [25]. We report here the isolation of n = 7/109 (6.4%) ESBL *E. coli* from Wallon meat lamb. Two phenotypical ESBL isolates showed atypical ESBL AST profiles and sequencing revealed that they harbored only additional beta-lactamase coding genes against penicillin, which most likely caused the ESBL phenotype. All but one *E. coli* isolate belonged to the phylogroup A and B1, which are considered as commensals. Yet, it was not possible to back-trace the presence of ESBL *E. coli* to the use of antibiotics on this farm. The presence of the same ESBL gene *bla_TEM-1_* suggested its fecal–oral spread among the lambs, impacting the intestinal *E. coli* population through horizontal gene transfer. However, the identification of different ESBL genes indicated that individual lambs could serve as a host for several resistance genes. Sequence type (ST) analysis indicated the dissemination of a few successful clones, as exemplified by the occurrence of the same ST in seven animals. All isolates also displayed resistance pheno- and genotypes to non-beta-lactam antibiotics, indicating vertical and/or horizontal spread of resistances.

### 4.2. Ovine Slaughter Hygiene Prevents Carcass Contamination with MDR E. coli

Mutton, especially premium cuts, is often consumed rare or medium rare, and was identified as a risk for contamination through ingestion of food-borne hazards from non-EU mutton samples, including *E. coli* O157:H7, β-lactamase-producing *P. mirabilis* and *Salmonella* [37,38,39]. While one STEC isolate was isolated in vivo, no ESBL or colistin-resistant *E. coli* were isolated from the meat lamb carcasses in our study. This is supported by findings from Italy, where none of the raw retail sheep meat harbored *mcr*-positive *E. coli* [40]. Conversely, Ghafur et al. reported the presence of 10% colistin-resistant bacteria in Indian mutton (n = 3/29) including an isolate of *E. coli* carrying *mcr-1* [41]. The stringent rules on slaughter hygiene in the EU aim at the highest level of consumer protection and are likely to have prompted the differences between the results from the EU and outside of the EU. Nevertheless, *E. coli* from raw cured mutton sausages were already identified as the etiological cause of a food-borne outbreak of hemolytic uremic syndrome in Norway [42]. Moreover, other raw products of ovine origin, such as 42.3% of Jordanian sheep milk samples carried phenotypically colistin resistant *E. coli* based on disk diffusion and 6.8% of milk samples were positive for *mcr-1* carrying *E. coli*, of which 33.3% were multi-drug-resistant [43]. The same authors also report the presence of similar isolates in cheeses made from raw sheep’s milk [44]. Sheep milk is predominantly used raw for cheese making and previous studies confirmed the presence of MDR *E. coli* as well in EU raw sheep milk cheese [45].

### 4.3. Influence of Husbandry Conditions on AMR Bacteria Carriage

No phenotypic colistin-resistant or ESBL *E. coli* were isolated from the sampled lambs under the organic scheme. Several factors are likely to have contributed to this difference, including the different husbandry conditions, hygiene and manure management practices between conventional and organic farming, which could have impacted the spread of bacteria during rearing. Conversely to the conventionally reared lambs, the organic lambs in our study had a very low stocking density and day-long outdoor access, which can foster the immune system, lessening their susceptibility to catching and carrying on pathogenic bacteria. While data on antimicrobial use (AMU) in sheep at the EU level are generally scarce, we tracked the AMU for all sampled lambs and none, neither the conventionally nor the organically reared lambs, received any antibiotic during their lifespan. However, 20% (n = 4/20) in 2020, resp., 10% (n = 1/10) in 2021 of the conventionally reared mother ewes received a macrolide antibiotic (6 mg/kg body weight gamithromycine) subcutaneously once in the month prior to delivery as foot rot treatment. The ewes under the organic scheme did not receive antibiotics during their gestation. While *E. coli* is intrinsically resistant to macrolide antibiotics due to their membrane impermeability, the foot rot treatment could have exacerbated selection pressure within the conventionally reared ewe population and consequently their lambs. Yet, our sample size was too limited to draw causal conclusions. A systematic review found, however, that better animal welfare leads to lower AMU in pigs, cattle and poultry [46]. Conversely, a positive association of AMU and AMR in animals and humans was shown before through the latest JIACRA report, indicating, for example, the use of aminopenicillins and fluoroquinolones in calves and resistance to them in *E. coli* from calves [9]. Previous studies demonstrated significantly lower AMR of *E. coli* originating from organic compared with conventional pig production for Denmark, France, Italy and Sweden [47]. Similar results were reported with significant differences for other bacteria such as *Campylobacter coli* in organic pigs from France and *Campylobacter jejuni* in organic turkey meat from Germany [48,49]. EU regulatory organic farming standards restrict the use of antibiotics and require firmer adherence to hygiene practices, emphasize animal welfare-friendly housing conditions and husbandry practices and respect for set stocking densities and minimum surfaces for indoor and outdoor areas [50]. In addition, the lambs under the organic scheme received solely organic feed and it was shown before that differences in feed quality and sourcing can influence the gut microbiota of animals, potentially affecting their susceptibility to colonization by pathogenic bacteria [51].

While the organic farmed meat lambs did not carry ESBL or colistin-resistant *E. coli*, risks for the commercialization of extensive rearing include irregularity in supply, carcass yield variability and organoleptic meat quality variations [52]. Due to the limited sample size, further research will be necessary to elucidate the true impact of husbandry conditions on AMR in meat lambs.

### 4.4. Limitations

This preliminary study is based on a limited sample size and no causal conclusion can be drawn from this comparison. In addition, the methodology focusing on selective agar and further characterization of only phenotypically ESBL or colistin-resistant isolates in a targeted approach excluded the detection and further characterization of phenotypically completely sensitive isolates to beta-lactam antibiotics and to colistin. Consequently, phenotypically completely sensitive isolates to beta-lactam antibiotics and colistin from the samples of this study may harbor other additional AMR genes, which remained undetected.

Our results obtained after CHROMID^®^ COL-R plate reading and MALDI-TOF identification indicated phenotypically colistin-resistant *E. coli* while the AST confirmed only reduced sensitivity to colistin with no strain surpassing an MIC of >2 μg/mL. Studies on the performance of CHROMID^®^ COL-R plates have shown sensitivity ranging from 86.8 to 99% and specificity ranging from 97 to 100% [53,54]. Due to the high specificity of this method, our isolates were initially not considered as false-positive. However, growth on CHROMID^®^ COL-R plates is considered positive when it occupies the entire plaque, whereas it is considered negative if colonies are only observed at the inoculation zone [55]. The broth microdilution method also has limitations, as colistin binds to the plastic of polystyrene trays, reducing the effective concentration in the broth and consequently altering the MIC values [56]. However, if isolates are at different ends of the sensitivity spectrum, the number of errors will be low, unlike isolates close to the critical threshold for which the number of errors will be higher [57].

AMRfinder is based on a protein-focused approach and allows for generally high prediction of up to 98% of positive correlations between pheno- and genotypes [58]. However, the discrepancies we observed may be due to several reasons, such as overproduction of resistance enzymes in the presence of the tested antibiotic. Notwithstanding, modifications to permeability or efflux may result in a decrease in the intracellular concentration of the inhibitor or the antibiotic itself, leading to reduced susceptibility. Yet, previous studies have demonstrated that clinical breakpoints, while crucial for guiding treatment, do not always align with the presence or absence of resistance genes in *E. coli* and *Salmonella* [59,60]. Furthermore, the nested hierarchical classification of AMR proteins into families allows AMRfinder to accurately name novel AMR genes, avoiding the risk of assigning incorrect functions through overly specific gene names. Without a clear understanding of the significance of similarity—rather than exact identity—to known AMR genes, relying on a ‘highest scoring hit’ approach can result in misleading conclusions about AMR gene content. Additional technical improvements will be required to reduce discrepancies per isolate, especially if clinical prediction is the primary objective [58].

## 5. Conclusions

The global interest in colistin resistance following the discovery of plasmid-borne *mcr* genes has led to a better understanding of the livestock reservoir, including products thereof. Our study showed a transient fecal carriage of *E. coli* with a reduced colistin sensitivity, thereof ESBL *E. coli* in two batches of commercially reared meat lambs. While colistin was never used in these batches, the reduced sensitivity was shown to be due to the chromosomal *pmrB* gene. From their carcasses, no ESBL *E. coli* with a reduced colistin sensitivity were isolated, indicating high slaughter hygiene and successful mitigation of the public health risk. It is imperative to further develop the prudent and judicious use of antimicrobials, including colistin and latest-generation cephalosporins in animals and humans alike to curb the global AMR burden in a One Health approach. Prevention of diseases through improved animal husbandry, including lower stocking density, resilient breeds, improved biosecurity and regular animal health visits, should be promoted to further reduce the need to use antimicrobials in animals and certification labels such as the organic scheme seem to serve this purpose. However, harmonized cross-sectorial AMR surveillance, including the sharing of phenotypic and genotypic data between countries, will be essential to monitor trends in minor animal species and assess the true extent of the public health risk.

## Figures and Tables

**Table 1:** Genetic characterization of seven *E. coli* isolates with reduced sensitivity to colistin from Wallon meat lamb fecal in vivo samples. ST: sequence type, NA: no available result.

Animal ID	Isolate	Pathovar	ST/ST Complex	Genes Conferring Colistin Resistance	Genes Conferring Fosfomycin Resistance
A2	6530-126	*E. coli*—STEC (*stx1* and *stx2*)	33/NA	*pmrB_Y358N*	*glpT_E448K*
A5	7632-127	NA	1246/NA	*pmrB_Y358N*	*glpT_E448K*
A6	7639-125	*E. coli*—EPEC (*eae*)	17/ST20 Cplx	*pmrB_Y358N*	*glpT_E448K*
A8	7674-128	*E. coli*—EPEC (*eae*)	17/ST20 Cplx	*pmrB_Y358N*	*glpT_E448K*
A9	7676-129	*E. coli*—EPEC (*eae*)	642/ST278 Cplx	NA	*glpT_E448K*
A16	7553-111	NA	345/ST23 Cplx	*pmrB_Y358N*	*glpT_E448K*
A20	7548-112	NA	761/ST10 Cplx	NA	NA

**Table 2:** ESBL isolates from meat lambs categorized based on their MIC (broth microdilution) to antibiotics as susceptible (S) or resistant (R) for ampicillin (breakpoint > 8 mg/mL), ceftiofur (breakpoint > 4 mg/mL), colistin (breakpoint 2 mg/mL), gentamicin (>4 mg/mL), tetracycline (>8 mg/mL) and sulfadimethoxine (>64 mg/mL).

Animal ID	Isolate	Ampicillin S-R MIC	Ceftiofur S-R MIC	Colistin S-R MIC	Gentamicin S-R MIC	Tetracycline S-R MIC	Sulfadi- Methoxine S-R MIC
A4	7579-39	R >16 mg/mL	S 1 mg/mL	S 0.5 mg/mL	S =<1 mg/mL	R >8 mg/mL	R >256 mg/mL
	7579-5	R >16 mg/mL	S 2 mg/mL	S 0.5 mg/mL	S 2 mg/mL	S 4 mg/mL	R >256 mg/mL
A9	7573-23	R >16 mg/mL	S 2 mg/mL	S 0.5 mg/mL	S =<1 mg/mL	S 4 mg/mL	R >256 mg/mL
A10	7572-8	R >16 mg/mL	R 8 mg/mL	S 0.5 mg/mL	S =<1 mg/mL	S 8 mg/mL	R >256 mg/mL
A11	7570-9	R >16 mg/mL	S 2 mg/mL	S 0.5 mg/mL	S 4 mg/mL	S 4 mg/mL	R >256 mg/mL
A14	7544-10	R >16 mg/mL	S 2 mg/mL	S 0.5 mg/mL	S =<1 mg/mL	S 4 mg/mL	R >256 mg/mL
A17	7575-12	R >16 mg/mL	S 2 mg/mL	S 0.5 mg/mL	S =<1 mg/mL	S 4 mg/mL	R >256 mg/mL
A20	7548-19	R >16 mg/mL	S 0.5 mg/mL	S 0.5 mg/mL	R >8 mg/mL	S 4 mg/mL	R >256 mg/mL
	7548-11	R >16 mg/mL	S 2 mg/mL	S 0.5 mg/mL	S =<1 mg/mL	S 4 mg/mL	R >256 mg/mL

**Table 3:** Characteristics of phenotypically extended-spectrum beta-lactamase-producing (ESBL)-producing *E. coli* isolates collected from Wallon meat lambs, ST: Sequence type.

Animal ID	Isolate	ST	ST Complex	Lineage	fimH (fimTyper)	Aminoglycoside	BL Inhibitor	Carbapenemase	Colistin	ESBL	Fosfomycin	Macrolide	Penicillin	Phenicol	Quinolone	Sulfonamide	Tetracycline	Trimethoprim	Lincosamide
A4	7579-39	10	ST10 Cplx	A	fimH54	*aadA1*, *aadA2*	-	-	-	-	-	-	*blaCARB-2*	*cmlA1*	*-*	*sul3*	tet(A)	*dfrA16*	*-*
A4	7579-5	3594	ST469 Cplx	AxB1	fimH31	*aadA2*	-	-	*pmrB_Y358N*	*blaTEM-52*	*glpT_E448K*	-	-	-	-	-	-	*-*	*lnu(F)*
A9	7573-23	3594	ST469 Cplx	AxB1	fimH31	*aadA2*	-	-	*pmrB_Y358N*	*blaTEM-52*	*glpT_E448K*	-	-	-	-	-	-	*-*	*lnu(F)*
A10	7572-8	3594	ST469 Cplx	AxB1	fimH31	*aadA2*	-	-	*pmrB_Y358N*	*blaTEM-52*	*glpT_E448K*	-	-	-	-	-	-	*-*	*lnu(F)*
A11	7570-9	3594	ST469 Cplx	AxB1	fimH31	*aadA2*	-	-	*pmrB_Y358N*	*blaTEM-52*	*glpT_E448K*	-	-	-	-	-	-	*-*	*lnu(F)*
A14	7544-10	3594	ST469 Cplx	AxB1	fimH31	*aadA2*	-	-	*pmrB_Y358N*	*blaTEM-52*	*glpT_E448K*	-	-	-	-	-	-	*-*	*lnu(F)*
A17	7575-12	3594	ST469 Cplx	AxB1	fimH31	*aadA2*	-	-	*pmrB_Y358N*	*blaTEM-52*	*glpT_E448K*	-	-	-	-	-	-	*-*	*lnu(F)*
A20	7548-19	301	ST165 Cplx		fimH902	*aadA2*, *aac(3)-IId*	-	-	-	-	*glpT_E448K*	*mph(A)*	*blaTEM-1*	*-*	*-*	*sul1*	*-*	*dfrA12*	*-*
A20	7548-11	3594	ST469 Cplx	AxB1	fimH31	*aadA2*	-	-	*pmrB_Y358N*	*blaTEM-52*	*glpT_E448K*	-	-	-	-	-	-	*-*	*lnu(F)*

## Data Availability

The original contributions presented in the study are included in the article/Supplementary Material, further inquiries can be directed to the corresponding author/s.

## Supplementary Materials

The following supporting information can be downloaded at: https://www.mdpi.com/article/10.3390/ani14203038/s1, Table S1: identification and analyses of the in vivo and ex vivo meat lamb samples, Table S2: characterization of ESBL-producing *E. coli* with reduced colistin sensitivity from Wallon meat lambs.
