# Supplementary material for: Conventionally Reared Wallon Meat Lambs Carry Transiently Multi-Drug-Resistant Escherichia coli with Reduced Sensitivity to Colistin Before Slaughter
Source: Animals (Basel). 2024 Oct 21;14(20):3038. doi: 10.3390/ani14203038 (PMC11505500; doi:10.3390/ani14203038)
Supplement: Supplementary file 1 [file animals-14-03038-s001.zip › Table S1 R3.pdf]

Table S1. Identification and analyses of the *in vivo* and *ex vivo* meat lamb samples

Lamb	Time	Month	Year	CHROMID® COL-R	CHROMID® ESBL	Identification MALDI/TOF
**A1**	T1	June	2020	-	-	
	T2	July	2020	-	-	
	T3	August	2020	-	-	
	T4	October	2020	-	-	
	C	October	2020	-	-	
**A2**	T1	June	2020	-	-	
	T2	July	2020	-	-	
	T3	August	2020	-	-	
	T4	October	2020	-	-	
	C	October	2020	-	-	
**A3**	T1	June	2020	-	-	
	T2	July	2020	-	-	
	T3	August	2020	-	-	
	T4	October	2020	-	-	
	C	October	2020	-	-	
**A4**	T1	June	2020	-	+	*E. coli*
	T2	July	2020	-	-	
	T3	August	2020	-	+	*E. coli*
	T4	October	2020	-	-	
	C	October	2020	-	-	
**A5**	T1	June	2020	+	-	*Providencia stuartii*
	T2	July	2020	-	-	
	T3	August	2020	-	-	
	T4	October	2020	-	-	
	C	October	2020	-	-	
**A6**	T1	June	2020	-	-	
	T2	July	2020	-	-	
	T3	August	2020	-	-	
	T4	October	2020	-	-	
	C	October	2020	-	-	
**A7**	T1	June	2020	-	-	
	T2	July	2020	-	-	
	T3	August	2020	-	-	
	T4	October	2020	-	-	
	C	October	2020	-	-	
**A8**	T1	June	2020	-	-	
	T2	July	2020	-	-	

	T3	August	2020	-	-	
	T4	October	2020	-	-	
	C	October	2020	-	-	
**A9**	T1	June	2020	-	-	
	T2	July	2020	-	-	
	T3	August	2020	-	+	*E. coli*
	T4	October	2020	-	-	
	C	October	2020	-	-	
**A10**	T1	June	2020	-	-	
	T2	July	2020	-	-	
	T3	August	2020	-	+	*E. coli*
	T4	October	2020	-	-	
	C	October	2020	-	-	
**A11**	T1	June	2020	-	-	
	T2	July	2020	-	-	
	T3	August	2020	-	+	*E. coli*
	T4	October	2020	-	-	
	C	October	2020	-	-	
**A12**	T1	June	2020	-	-	
	T2	July	2020	-	-	
	T3	August	2020	-	-	
	T4	October	2020	-	-	
	C	October	2020	-	-	
**A13**	T1	June	2020	-	-	
	T2	July	2020	-	-	
	T3	August	2020	-	-	
	T4	October	2020	-	-	
	C	October	2020	-	-	
**A14**	T1	June	2020	-	-	
	T2	July	2020	-	-	
	T3	August	2020	-	+	*E. coli*
	T4	October	2020	-	-	
	C	October	2020	-	-	
**A15**	T1	June	2020	-	-	
	T2	July	2020	-	-	
	T3	August	2020	-	-	
	T4	October	2020	-	-	
	C	October	2020	-	-	
**A16**	T1	June	2020	+	-	*Providencia stuartii*

	T2	July	2020	+	-	*E. coli*
	T3	August	2020	*No sample (unintentionally omitted during sampling)*		
	T4	October	2020	-	-	
	C	October	2020	-	-	
**A17**	T1	June	2020	-	-	
	T2	July	2020	-	-	
	T3	August	2020	-	+	*E. coli*
	T4	October	2020	-	-	
	C	October	2020	-	-	
**A18**	T1	June	2020	-	-	
	T2	July	2020	-	-	
	T3	August	2020	-	-	
	T4	October	2020	-	-	
	C	October	2020	-	-	
**A19**	T1	June	2020	-	-	
	T2	July	2020	-	-	
	T3	August	2020	-	-	
	T4	October	2020	-	-	
	C	October	2020	-	-	
**A20**	T1	June	2020	-	+	*E. coli*
	T2	July	2020	-	-	
	T3	August	2020	+	+	*E. coli*
	T4	October	2020	-	-	
	C	October	2020	-	-	
**2021**						
**Lamb**	**Time**	**Month**	**Year**	**CHROMID® COL-R**	**CHROMID® ESBL**	**Identification MALDI/TOF**
**A1**	T1	January	2021	-	-	
	T2	March	2021	-	-	
	T3	April	2021	-	-	
	C	April	2021	-	-	
**A2**	T1	January	2021	-	-	
	T2	March	2021	-	-	
	T3	April	2021	+	-	*E. coli*
	C	April	2021	-	-	
**A3**	T1	January	2021	-	-	
	T2	March	2021	-	-	
	T3	April	2021	-	-	
	C	April	2021	-	-	
**A4**	T1	January	2021	-	-	

	T2	March	2021	-	-	
	T3	April	2021	-	-	
	C	April	2021	-	-	
**A5**	T1	January	2021	-	-	
	T2	March	2021	-	-	
	T3	April	2021	+	-	*E. coli*
	C	April	2021	-	-	
**A6**	T1	January	2021	-	-	
	T2	March	2021	+	-	*E. coli*
	T3	April	2021	-	-	
	C	April	2021	-	-	
**A7**	T1	January	2021	-	-	
	T2	March	2021	-	-	
	T3	April	2021	-	-	
	C	April	2021	-	-	
**A8**	T1	January	2021	-	-	
	T2	March	2021	-	-	
	T3	April	2021	+	-	*E. coli*
	C	April	2021	-	-	
**A9**	T1	January	2021	-	-	
	T2	March	2021	-	-	
	T3	April	2021	+	-	*E. coli*
	C	April	2021	-	-	
**A10**	T1	January	2021	-	-	
	T2	March	2021	-	-	
	T3	April	2021		-	
	C	April	2021		-	

T1-T4: Rectal swabs; C: Carcass swabs;
+ : presence of presumtive *E. coli* colonies
- : absence of presumtive *E.coli* colonies
